# Sepsis: Immunopathology, Immunotherapies, and Future Perspectives

**DOI:** 10.5152/eurasianjmed.2022.22314

**Published:** 2022-12-01

**Authors:** Taha Tavaci, Nurullah Akgun

**Affiliations:** 1 Ataturk University, Faculty of Medicine, Department of Pharmacology, Erzurum, Turkey

**Keywords:** Sepsis, immunotherapy, immunopathology

## Abstract

Sepsis is a syndrome that includes physiological, pathological, and biochemical abnormalities resulting from the host immune response to infection. Despite the improved treatment modalities in recent years, the incidence and mortality of sepsis are still increasing. Sepsis immunopathology is increasingly attracting the attention of researchers. The successes experienced with immunotherapeutics in the treatment of cancer and coronavirus disease 2019, which are diseases with similar pathophysiological features and common immune defects with sepsis, have given rise to the hope that similar successes can be achieved in the treatment of sepsis. In this review, future perspectives on the immunopathology of sepsis and immunotherapeutics are presented to improve the current understanding of the disease.

**Main Points**

- The failure of many clinical trials in sepsis has required new insights into the pathophysiological basis of the disease.
- Detailed examination of sepsis immunopathology has strengthened our understanding of sepsis pathogenesis.
- The success achieved with immunotherapy in coronavirus disease 2019 and cancer treatments has also been hope for sepsis.

## Introduction

Sepsis is not merely a modern problem. The condition was referenced in poems dating back thousands of years.^1^ According to Avicenna, it was the decay of blood and tissues with fever.^2^ Current definitions explain it to be a life-threatening organ dysfunction caused by a systemic, dysregulated inflammatory host response to infection.^3-6^

Despite developing treatment modalities and a better understanding of the pathophysiology of sepsis, its mortality is extremely high due to tissue damage, vital organ failures, and excessive inflammatory responses.^7-16^ In addition, the morbidity of post-sepsis with its wide spectrum of symptoms constitutes a severe health problem.^17^ Sepsis is a huge burden on health economics due to high treatment costs.^18^ With an alarmingly high rate of incidence, sepsis has become one of the leading causes of death in the world. Half of all in-hospital deaths in the USA are directly or indirectly related to sepsis.^19^ Sepsis has undoubtedly transformed into an issue in global public health.^20^ In 2017, sepsis was officially recognized as a global health priority by the World Health Assembly.^21^

Sepsis is a syndrome that requires urgent treatment. Based on recent developments, both the rate of early diagnosis and the rate of treatment have increased.^22^ An effective sepsis treatment is as important as early sepsis diagnosis. Although nosocomial deaths due to sepsis have decreased in recent years with the onset of supportive care and early diagnosis, our improved understanding of its pathogenesis has not greatly improved outcomes.^23^

Consequently, a re-examination of the pathophysiological basis underlying sepsis has become necessary.^15^ Researchers have done a great deal of work on its immunopathology in the last decade.^14,15^ The complex pathophysiology of sepsis has become better understood, given new theories circulating on issues regarding host immune response.^15^ Understanding the mechanisms responsible for pro- and anti-inflammatory responses in the disease has shed light on effective treatment approaches.^15^

There is no known, effective pharmacological treatment for sepsis.^21^ The researchers chose to call it a pharmaceutical graveyard because no effective treatment for sepsis could be found.^24^ Although successes have been achieved in sepsis experiments with various drugs in preclinical animal experiments,^16,25-39^ the clinical translation of these successes has been almost nonexistent.^30^

Sepsis and cancer share many pathophysiological features and common immune defects.^15,31^ The achievements that immunotherapy has had in cancer treatment, especially in this period when the immunology of sepsis was well understood, gave rise to hope that similar successes can be attained against sepsis.^15^ The potentiality of overcoming the failures experienced in clinical trials for sepsis^32^ with sepsis immunotherapy is extremely attractive. In order to identify new drug targets, there is a need to increase the focus on basic studies of the pathophysiology of the disease.^32^ This review aims to contribute to the future perspective by examining the immunopathology of sepsis and immunotherapies.

## Pro-Inflammatory Mechanisms in Sepsis

An excessive pro-inflammatory response to the pathogen is the hallmark of sepsis, a disease that is almost synonymous with terms such as excessive inflammation, cytokine storm, and systemic inflammation.^6,33,34^ In sepsis, the eradication of pathogens is targeted with the pro-inflammatory response, and leukocyte activation, cytokine production, reactive oxygen species release, and complement and coagulation system activation occur with the pro-inflammatory response.^35^ An uncontrolled, excessive pro-inflammatory response can have detrimental effects on the host, such as high fever, tachycardia, tachypnea, hypotension, coagulation disorders, and organ failure from collateral tissue damage.^36^ After infection, the sepsis agent encounters the host’s innate immune system, which becomes alert to pathogens by recognizing pathogen-associated molecular patterns (PAMPs) through pattern recognition receptors.^37^ Pattern recognition receptors are also capable of distinguishing endogenous distress signals called damage-associated molecular patterns (DAMPs). These signals are released by damaged/necrotic host cells and potentiate the pro-inflammatory response.^38^

Neutrophils play an important role in controlling the infection. However, it is thought that neutrophil migration and antimicrobial activity are impaired in sepsis and contribute to the dysregulation of immune responses.^39^ It was, nevertheless, observed that neutrophil lifespan increased with the activation of anti-apoptotic pathways during sepsis.^40^ One of the numerous antimicrobial mechanisms of neutrophils is neutrophil extracellular traps (NETs), consisting of modified chromatin “decorated” with bactericidal proteins.^41^ Vascular inflammation and coagulation are enhanced by the release of NETs.^42^ However, NETs can cause also tissue damage. An association has been found between increased NETs and organ dysfunction in sepsis patients.^43^ Cytokines, such as tumor necrosis factor-α (TNF-α), interleukin (IL)-1β, and IL-6, are important mediators of the innate immune system and play a crucial role in the first response to injury or infection. Pathogen-associated molecular patterns or DAMPs from invading organisms are recognized by macrophage receptors such as Toll-like receptors. As a result, the production of pro-inflammatory cytokines such as TNF-α, IL-1β, and IL-6 and chemokines such as IL-8 and monocyte chemoattractant protein-1 occurs. Tumor necrosis factor-α, IL-6, IL-1β, IL-12, nuclear factor kappa B, and IL-18 are pro-inflammatory cytokines that have their own impact on sepsis inflammation. Their functions have been repeatedly demonstrated in many experimental studies.^34,38,44^

The complement system is a very powerful component of immunity. However, uncontrolled complement activation can harm the host. It has been shown in experiments that the results of sepsis are improved by blocking the complement component 5a signal.^45^ The coagulation system is also strongly activated during the onset of the disease. This response is likely based on a reflex that creates microvascular occlusion to prevent the spread of the pathogen into the system. Platelets increase immune cell activation and inflammation, facilitate vaso-occlusive thrombus formation in capillary vessel beds, and may have direct toxic effects on cells. Excessive platelet activation likely contributes to organ damage.^46^ The excessive inflammatory response in sepsis affects all organs and tissues as well as activates the endothelium, causing the release of pro- or anti-inflammatory mediators. The integrity of the endothelial barrier also becomes compromised. Increased barrier insufficiency causes leakage of intravascular proteins and plasma into the extravascular space.^47^ While this infiltration provides benefits in infected areas by the entry of complement, immunoglobulins, and other protective molecules, it often causes diffuse tissue edema and reduced microvascular perfusion.^14^ Oxidative stress occurs in response to hypoxia, microbial clearance, and endothelial repair processes in sepsis. However, as a result of an excessive increase in reactive oxygen species, the balance of the antioxidant system is disrupted and endothelial damage inevitably follows. It is accepted that reactive oxygen species play an important role in triggering many mediators and pro-inflammatory cytokines produced in acute inflammatory responses associated with sepsis.^48^

## Immunosuppression Mechanisms in Sepsis

When examining host response theories regarding sepsis, anti-inflammatory responses are a vital element of its pathophysiology.^15^ Lymphocyte exhaustion can be defined as progressive loss of functionality and decreased proliferative ability induced by prolonged antigen stimulation in the course of cancer or chronic infections.^49^ A strong depletion of CD4+ and CD8+ T cells, B cells, and dendritic cells is seen in sepsis.^50^ T lymphocyte exhaustion was observed in postmortem examinations of patients who died from the disease. In many fatal cases, increased expression of programmed cell death 1 (PD1) was observed in CD4+ T cells.^50^ Apoptosis aims to remove damaged cells and maintain homeostasis under normal physiological conditions.^51^ In the sepsis experiment performed with the cecal ligation and puncture model in mice, it was shown that Bim, caspase-3, caspase-8, and caspase-9 levels were significantly elevated, thus increasing apoptosis in sepsis.^52^ Antigen-presenting cells (APCs) are derived in the bone marrow. This team consists of dendritic cells, Langerhans cells, macrophages, and B cells. Antigen-presenting cells capture antigens and process and present antigens to T cells.^53^ Sepsis indirectly or directly impairs the function of almost all immune cells, as well as impairing the functions of APCs, preventing them from fully performing their functions.^14,15^

There are a number of immune regulatory molecules that control the pro-inflammatory cytokine response. Chief among these are anti-inflammatory cytokines. Major anti-inflammatory cytokines include IL-4, IL-10, IL-11, and IL-13.^54,55^ Interleukin-4, IL-10, and IL-37 are anti-inflammatory cytokines that have important functions, especially in sepsis.^56^ Interleukin-4 and IL-10 inhibit the differentiation of CD4+ T cells into T helper 1 cells, reducing the release of pro-inflammatory cytokines, including IL-2 and interferon-γ (IFN-γ). Interleukin-10, an immunosuppressive cytokine with multiple functions, is mainly secreted by monocytes/macrophages and T helper 2 cells. Interleukin-10 has been shown to inhibit TNF formation in sepsis-induced mice. Interleukin-10 can also promote the proliferation of myeloid-derived suppressor cells in mice with sepsis and exacerbate immunosuppression in mice with advanced sepsis.^56^ Although belonging to the IL-1 family, which contains cytokines that generally have pro-inflammatory properties, IL-37 reduces inflammation and adaptive immune responses. Interleukin-37 can also inhibit the release of pro-inflammatory cytokines.^57^ Expression of IL-37 in patients with sepsis is significantly upregulated, which may obstruct proliferation and release of pro-inflammatory cytokines, and is closely related to the severity of sepsis-induced immunosuppression.^58^

## Immunotherapies in Sepsis

The search for treatments that specifically target the immune system has dominated the sepsis research field for over 40 years.^59^ Granulocyte-macrophage colony-stimulating factor (GM-CSF), recombinant human IL-7, IFN-γ, PD1 and programmed cell death protein 1 (PDL1)-specific antibodies, anti-TNF-α, recombinant human IL-3 and IL-15 treatments are among those that have undergone significant research.

It is known that dysregulated immune responses triggered by sepsis cause dysfunction of neutrophils.^60^ Granulocyte-macrophage colony-stimulating factor enhances immunity by augmenting the bactericidal abilities of neutrophils and monocytes during sepsis.^15^ According to the results of a clinical trial with GM-CSF, GM-CSF treatment helped shorten the duration of antibiotic therapy, although it did not change the mortality rate.^61^ Other studies have shown that patients treated with GM-CSF in the immunosuppressive phase of sepsis shorten the ventilator-dependent time and intensive care periods.^62^ Interleukin-7 is a pleiotropic cytokine and is extremely important for the development of T cells.^63^ Considering the T cell exhaustion that develops in sepsis, the importance of IL-7 in treatment is better understood. In clinical trials, recombinant IL-7 has been used to treat idiopathic lymphopenia and diseases caused by lymphopenia, proving its potential for the future.^64^ In a study of mice with sepsis, IL-7 treatment increased the percentage of survival.^65^ In phase II clinical study, it was shown that 27 septic shock lymphopenia patients did not develop excessive inflammatory reactions or experience worsened organ dysfunction as a result of IL-7 treatment, but significantly saw increased CD4+ and CD8+ T lymphocyte counts.^66^

In a study on the therapeutic effect of IFN-γ in sepsis patients, it was observed that monocyte human leukocyte antigen-DR isotype expression accelerated and TNF-α secretion from monocytes increased, thereby improving pathogen elimination capacity.^67^ If IFN-γ is added to the treatment protocol in immunosuppressed patients, patients exhibiting adaptive immune dysfunction or chronic inflammation, or at risk, its effects on sepsis can be seen more clearly.^68^ Programmed cell death 1 is a protein expressed in T cells, and when it binds with PDL1, it prevents other cells from being killed by T cells.^69^ It has been suggested that both anti-PD1 and anti-PDL1 treatments, which show great promise in cancer treatments, also have potential in sepsis-induced immunosuppression.^70^ The PD1–PDL1 blockade improved survival outcomes in animal models of bacterial sepsis.^71^ In a clinical patient study, the tolerability of an anti-PD1 antibody, nivolumab, was appropriate and did not cause conditions such as cytokine storm.^72^

Elevated TNF-α levels detected in the serum of septic patients in early sepsis studies led to the assumption that this cytokine has an important role in septic shock.^73^ Administration of neutralizing anti-TNF monoclonal antibodies to baboons 2 hours before induction of sepsis with *Escherichia coli* has been observed to protect animals from shock and organ failure.^74^ However, clinical studies conducted in the 90s resulted in the failure of TNF inhibition treatments.^75-77^ Interleukin-3 and IL-15 should also be considered as potential sepsis treatments. Being foundational in the development and activation of effector and memory T, Natural killer and Natural killer T cells, and neutrophils, IL-15 has great potential in the treatment of sepsis immune dysfunction.^78^ In fact, an increased survival rate has been noted in mice with sepsis treated with IL-15.^79^ The synergistic role of IL-3 with IL-7 makes it a candidate therapeutic to augment the potential effect of IL-7.^80^

## Future Perspectives

Although excessive inflammation is one of the trademarks of sepsis, the terms sepsis-induced immune dysfunction and immunoparalysis have gained traction in the last 2 decades.^15,59,81,82^ This shows that the cult and plain excessive inflammatory view of sepsis has changed. While it is argued that mortality in sepsis is due to inflammation and permanent immune activation, immunosuppression is increasingly recognized as the driving force behind sepsis mortality.^24,68,83^

A few decades ago, treatment strategies for sepsis were almost entirely aimed at suppressing the hyperinflammatory response in the early stages.^59^ Clinical trials of anti-inflammatory therapies targeting specific inhibition of elements that cause excessive inflammation in sepsis are fraught with failures.^14^ In recent years, therapeutic strategies for immunosuppression in sepsis have also been developed. Some researchers also advocate for the use of immunostimulants in sepsis.^15^

Immunomodulation is revolutionizing the treatment of cancer, autoimmune diseases, and many other inflammatory disorders.^84^ Immunomodulatory therapy is very important in sepsis because patients may arrive in the early hyperinflammatory phase or in the immunosuppression phase.^15^ Whichever it is, deducing the phase is extremely important. While the sepsis agent is actively spreading through the body, anti-cytokine drugs may put the patient at a disadvantage in the fight against the agent. However, while targeting immunomodulation, immune augmentation in a sepsis patient with cytokine storm can take excessive inflammation to incredible levels.

Another issue is the effects of cytokine storm on the patient. If the clinician is monitoring patients with biomarkers that rise during the cytokine storm, there are certain factors to consider, as anti-inflammatory signal pathways are triggered in response to high inflammation in sepsis.^15^ Although we know how and the rate by which cytokines such as IL-6 bind to their receptors in the presence of specific antibodies such as tocilizumab,^85^ we still do not know exactly how cytokines bind to their receptors in sepsis. A second issue is that a biomarker followed in a patient with sepsis may show high levels in the blood, but the patient may not have any clinical symptoms. Hypothetically, in the presence of a cytokine receptor that can become desensitized with high cytokine values, the signal pathway may not be activated. On the other hand, the cause of cytokine elevation may also be a receptor in sensitivity itself. For this reason, it is extremely important to measure the activity of signaling pathways involved in inflammatory signaling in sepsis patients. In the treatment of sepsis, a treatment based on time-dependent correlations of organ damage with biomarker measurements and knowing the activity of the relevant inflammatory signaling pathway may be much more beneficial for patients.

In treatment, the principle of right medication, right dose, right patient, right time, and right route is extremely important.^86^ Unfortunately, finding the right time is almost impossible in sepsis patients. For this reason, the progression of clinical findings in patients will be important. In order to choose the right time for sepsis treatment, the clinician must first know which stage of sepsis the patient is in. However, in order to find the right drug expression in sepsis, the expression of the right time must be fulfilled. Examination of the molecular pathophysiology of the disease at short-term intervals during the sepsis process can provide important information. In this way, more accurate predictions can be made about which stage of sepsis the patient is in and how the host immune response may progress in sepsis.

Sepsis and cancer share many common immune defects.^15^ At the root of both diseases is the inability of the host’s immune system to cope with the initial insult. Immune dysfunctions due to sepsis and cancer are some of the common aspects of changes in the function of immune elements.^31^ Because of the major role of immunity in anti-tumor surveillance, scientists are investigating this issue, as latent malignancies are likely to be present in the host due to the strong acute inflammatory response or immune defects seen in patients with sepsis.^31,87^ Clinical and experimental data indicate a potent immunomodulatory effect of sepsis on cancer.^31^ The success of immunotherapies in cancer treatments has been well-proven in recent years.^88^ While cancer and sepsis have so many immunological points in common, the success rate seen in cancer immunotherapies has given rise to the hope that such treatment may also prove effective against sepsis. Though it is early now, in the future, sepsis may even be interpreted as “acute form of cancer.”

Sepsis immunotherapies are promising.^15^ Coronavirus disease 2019 (COVID-19) has encouraged us in this regard. The common features found in the immunopathogenesis and pathophysiology of sepsis and COVID-19, and the treatment management of COVID-19 benefited from the experience of sepsis.^89^ Immunotherapeutics such as tocilizumab and anakinra have been used successfully in combating COVID-19.^90,91^ This success is likely to be achieved in sepsis, which has many common pathophysiologic properties with COVID-19. Sepsis studies need a new direction. Here, the bedside and the bench need to work together. A molecular pathophysiology study that will examine the sepsis process in very thin sections in large patient populations will provide important information about how sepsis progresses and the optimal timing of treatment.
